# Targeted Radionuclide Therapy of Human Tumors

**DOI:** 10.3390/ijms17010033

**Published:** 2015-12-28

**Authors:** Sergey V. Gudkov, Natalya Yu. Shilyagina, Vladimir A. Vodeneev, Andrei V. Zvyagin

**Affiliations:** 1 Laboratory of Optical Theranostics, Lobachevsky Nizhny Novgorod State University, Gagarin Ave. 23, Nizhny Novgorod 603950, Russia; nat-lekanova@yandex.ru (N.Y.S.); v.vodeneev@mail.ru (V.A.V.); andrei.zvyagin@mq.edu.au (A.V.Z.); 2 Institute of Theoretical and Experimental Biophysics, Russian Academy of Sciences, Institutskaya St, 3, Pushchino, Moscow 142290, Russia; 3 Prokhorov Institute of General Physics, Russian Academy of Sciences, Vavilova St, 38, Moscow 119991, Russia; 4 ARC Centre of Excellence for Nanoscale BioPhotonics (CNBP), Macquarie University, Sydney 2109, Australia

**Keywords:** radio-immunotherapy, radionuclide, targeted therapy, α-emitter, β-emitter, Auger electron, antibody, peptide

## Abstract

Targeted radionuclide therapy is one of the most intensively developing directions of nuclear medicine. Unlike conventional external beam therapy, the targeted radionuclide therapy causes less collateral damage to normal tissues and allows targeted drug delivery to a clinically diagnosed neoplastic malformations, as well as metastasized cells and cellular clusters, thus providing systemic therapy of cancer. The methods of targeted radionuclide therapy are based on the use of molecular carriers of radionuclides with high affinity to antigens on the surface of tumor cells. The potential of targeted radionuclide therapy has markedly grown nowadays due to the expanded knowledge base in cancer biology, bioengineering, and radiochemistry. In this review, progress in the radionuclide therapy of hematological malignancies and approaches for treatment of solid tumors is addressed.

## 1. Introduction

Throughout its entire history the human race desired to hit the target with high precision. In the beginning this aim was realized though the invention of a sling, then with the help of bow and arrows, firearms, high-precision rocket weapons, lasers, *etc.* The target moved farther and became smaller, but it just forced the human intelligence to invent more complicated and accurate systems. Another evolutionary approach was the invention of bombs. This strategy does not necessarily require precision, the power is more important; the target is hit anyway, despite the great collateral damage. The radiotherapy of malignancies also includes both of these approaches, and radiation therapy can be compared to bombs, affecting healthy tissues in one way or another. The weapon of high precision here is targeted radionuclide therapy (TRNT), which is the topic of the current review.

**Figure 1:**
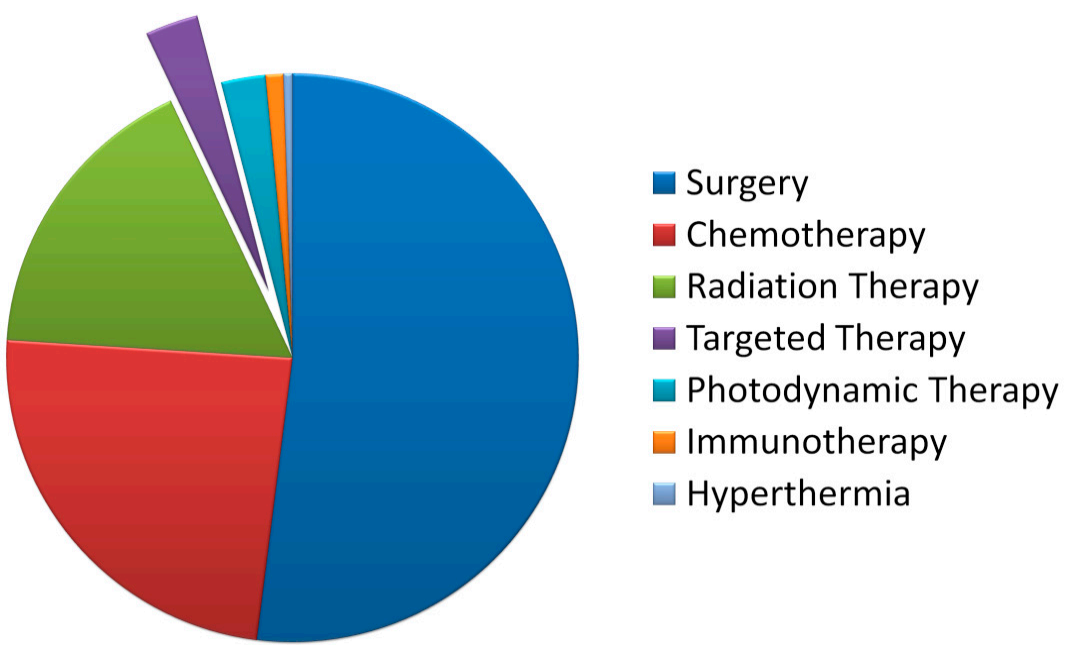
A pie chart of the prevalence of cancer treatments.

The targeted radionuclide therapy is based on the use of high-affinity molecules as carriers of radionuclides to tumor cells [1]. Pharmaceuticals for targeted radionuclide therapy are often injected intravenously or intracavitary. Following the injection, such drugs enter the blood stream and eventually reach their target—a target molecule on the surface of tumor cells. A radionuclide attached to the pharmaceutical directly interacts with the rumor cell [2]. Cursory analysis of the existing clinical practices shows that, presently, TRNT occupies several percents of the total number of the clinically-used procedures, as presented in Figure 1, which is translated into the value of sub-million cases, considering more than 14 million oncology patients registered annually.

In clinical practice, TRNT is widely applied in the treatment of the most radiosensitive tumors, particularly to leukemias and lymphomas. Solid tumors are generally more radio-resistant and sometimes require an order-of-magnitude greater doses of ionizing radiation. The relative radiosensitivity of cancer cells normally correlates with that of normal tissue, from which the tumor is originated. Tissues with the higher radiosensitivity, for instance, red bone marrow, produce radiosensitive tumors, and conversely, radio-resistant tumors originate from radio-resistant tissues, such as the brain [3]. Regardless of the initial radiosensitivity, the main objective of the TRNT is the delivery of radionuclides to cancer cells without any risks for normal tissues, exposure of these cells to highly absorbed doses of ionizing radiation and their damage. At the present time, the development of novel spatial visualization methods for the assessment of absorbed dose both in tumors and normal tissues upon application of TRNT allows avoiding the side effects and toxicity from excessive irradiation, leading to personalization of the treatment regimen for every individual patient. This integration of therapy and diagnostics is a fundamental example of the possibilities of a theranostics approach, when a pharmaceutical is used for diagnostics (in trace amount), as well as for therapeutic purposes [4]. The clinical practice often involves the application of quantitative high-resolution positron emission tomography (PET) or computed tomography (CT) in order to realize the theranostic approach in targeted radionuclide therapy. Such a paradigm provides clinicians with a precise tumor topography and spatial dosimetry, making it possible to develop the most optimal personalized treatment regimen.

## 2. Features of Targeted Radionuclide Therapy

The method of TRNT is based on the selective accumulation of radionuclide-containing pharmaceuticals only in tumorous tissues. Ideally, TRNT should directly affect only cancer cells evading influence on normal cells. This requirement provides an opportunity to create a pharmaceutical with a high or—ideally—infinitely high therapeutic index, affording to acquire high efficiency with minimal health risks. In practice, this ideal case is virtually impossible, since slight damage to normal tissues occurs in the process of transportation to the target due to the bystander effect and catabolism of the pharmaceutical accompanied by a release of radionuclide [5]. Another advantage of the TRNT is patient convenience. Pharmaceuticals for targeted radionuclide therapy are injected within several minutes and subsequently affect the tumor, whereas the patient does not need to go through additional procedures and get more injections during this period of time [6]. The creation of such pharmaceuticals, possessing a prolonged effect and high therapeutic index, requires a careful selection of radionuclide along with a platform for its delivery into tumor.

## 3. Radionuclide Selection

It is obvious that radionuclides for targeted therapy must comply with several requirements.

**Figure 2:**
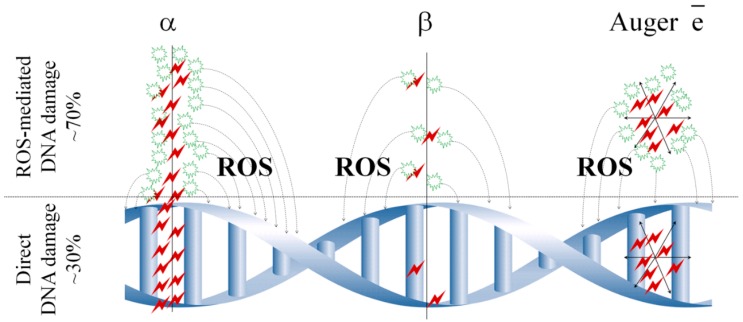
Schematic representation of ionization density along the path of α-, β-particles, and Auger electrons (α-particles are considered densely-ionizing radiation, β-particles are sparsely ionizing, and Auger electrons form clusters with a high density of ionization).

(1) The radioactive decay of radionuclides is expected to cause significant damage to cancer cells. Particulate radiation from the decaying radionuclide has much lower probably to damage biomolecules. This damage is mediated by reactive oxygen species (ROS) generated as the result of the radiolysis of water. Potentially, α-emitters, β-emitters, and Auger electron emitters are assumed to exhibit the highest therapeutic efficiency, because these types of radiation have the greatest relative biological effectiveness, *i.e.*, provide stronger destruction to biological systems at a given dose, in comparison to ionizing electromagnetic types of radiation (X-rays and γ-radiation) (Figure 2) [7]. However, Auger electron emitters appeared to be less effective compared to α- or β-emitters in clinical trials [8].

Linear energy transfer for α-particles is ~80 keV/μm; for β-particles, 0.2–2.0 keV/μm; for Auger electrons, 4–26 keV/μm. α-particles cause irreversible DNA damage and induce cell death if 2–3 tracks transit the cross-section of the DNA structure, while it requires 10^2^–10^3^ tracks for β-particles. The energy of β-particles has a more serious impact on the efficiency of therapy. This parameter should be tailored for treatment specific types of cancers via the choice of suitable radionuclides. For example, it is preferable to use the low-energy particles (less than 1 MeV) in case of treatment of leukemias, lymphomas, and metastases, whereas the use of β-particles with the high energy (>1 MeV) is beneficial for treatment of solid tumors [9].

(2) The choice of the irradiation type depends on tumor size and heterogeneity, as well as the inhomogeneity of the radionuclide distribution, pharmacokinetics, *etc.* [10]. The β-particle path length in biotissues can exceed ten millimeters, which underpin their rational use for relatively small tumors. The path length of α-particles in biotissues is in the range of 50–100 μm, making them most efficient against moderate-size neoplasms and micrometastases. Radionuclides emitting electrons due to the Auger effect are generally effective only when their carrier molecules can penetrate through the cell membrane and reach the nucleus (for example, using a compound ^111^In-Octreotide).

(3) Radionuclide half-life should correspond to pharmacokinetics of the carrier *in vivo*. This means that the half-life must be longer (although not much longer) than the time required for the preparation of pharmaceutical, its delivery to clinic, injection, and localization in a tumor. An optimal half-life for isotopes used for radionuclide therapy ranges from six hours to seven days [11]. The only exception is the isotope ^89^Sr having a half-life of 50 days [12]. More short-lived isotopes are inconvenient in terms of delivery to the site of therapy and storage, while radionuclides with longer life span promote an increase of absorbed dose not only in the target tissues, but also in the surrounding areas. Upon that, patients who were injected with long-lived radionuclides, for instance, the already mentioned ^89^Sr with the half-life period of 50 days, can themselves become a source of radiation hazard, underlying the necessity of their isolation, as well as organization of maximum security in clinical facilities, including isolated systems of water disposal and purification.

In clinical practice, the complete life span (*T*) is determined not only by the half-life of an isotope (*T_i_*), but also by the half-clearance time of an agent from the organism or target organ (*T_b_*). [13]. In general, the pattern is described by the formula: 1/*T* = 1/*T_i_* + 1/*T_b_*. The half-life of an isotope (*T_i_*) is a tabular value. The half-clearance time of a drug from the organism or target organ (*T_b_*) is largely due to the delivery system of choice, assuming the necessity of experimental studying of clearance dynamics for each agent. Analysis of dynamics often involves the short-lived gamma-emitting radionuclides, which are attached to carrier molecules instead of beta- or alpha-emitting radionuclides used in TRNT [14].

(4) Isotopes used for TRNT ought to have a high grade of chemical purity and free from trace impurities of the concomitant elements and metals. In particular, metal impurities inhibit the process of ”labeling” of carrier molecules with metal radionuclides.

(5) When therapy is accompanied by diagnostics, visualized dosimetry measurements and treatment response monitoring can be efficiently realized with the use of γ-emitting radionuclides. Ideally, γ-radiation is characterized by the low energy (between 70 and 300 keV, with an optimum around 140 keV), so as to ensure an effective activity of the γ-ray chamber and minimize the adverse effects on normal tissues [15].

(6) The ability of a radionuclide to bind to a wide variety of carrier molecules belonging to different chemical classes is an important property. The resulting pharmaceutical should be stable during short-term storage, as well as upon contact with biological liquids.

(7) Large-scale production of radionuclides for targeted therapy ought to be organized in a cost-effective manner. Extremely expensive radionuclides, even those having attractive characteristics, can hardly be widely applied.

This is due to the fact that to the cost of a radiopharmaceutical is to a large extent determined by the cost and availability of obtaining of its main component—radioactive isotope. The main sources of radionuclides are nuclear reactors, accelerators and radionuclide generators. In the global production of radionuclides a significant part is realized with the use of charged particle accelerators, most of which are cyclotrons of different types. Historically this fact is usually linked with a large number of research accelerators and their availability in the first years of the development of nuclear medicine at the turn of the 1940s and 1950s, as well as with a relatively low cost of production of most radionuclides. It should be noted that the production of some types of radionuclides requires a cyclotron with unique technical characteristics. For instance, ^211^At can be obtained only in 30 cyclotrons in the world [16]. An alternative approach for obtaining radionuclides assumes generators of short-lived radionuclides. This technology is based on the use of relatively long-lived isotopes (half-lives of months and years, like ^90^Sr with a half-life of 28 years), and their radioactive decay yield the formation of a daughter isotope, which has a therapeutic importance because of the short half-life (for example, ^90^Y with a half-life of 64 h) and stability of the resulting fallout. Daughter isotopes are occasionally derived from the generator by chemical extraction [17]. Long-lived radionuclides used in generators are produced in nuclear reactors, and the deriving of some isotopes also requires nuclear reactors with unique technical characteristics. For instance, ^188^W, whose daughter isotope is ^188^Re, can be obtained only in 2–3 reactors in the world [18].

The major features, such a half-life, decay nature, power, and method of producing, of radionuclides most frequently used today for targeted radionuclide therapy are presented in Table 1. Beta-emitting radionuclides ^131^I and ^90^Y are used in the clinical practice in approximately 90% cases of TRNT.

**Table 1:** Radionuclides applied in targeted radionuclide therapy.

Radionuclide	*T*_1/2_, h	*E*_max_, MeV (*)	Method of Producing
^124^I	100.1	β − 1.6 (~90%); 2.2 (~10%)	cyclotron
^131^I	192.0	β − 0.7 (89%); γ − 0.4 (82%); β/γ ** = 1	nuclear reactor
^86^Y	14.7	β − 1.2 (~90%); 1.6 (~10%)	cyclotron
^90^Y	64.8	β − 2.2 (100%)	generator ^90^Sr→^90^Y
^177^Lu	160.8	β − 0.5 (100%)	nuclear reactor
^188^Re	17.0	β − 2.0 (100%)	generator ^188^W→^188^Re
^64^Cu	12.7	β − 0.65 (61.5%), β − 0.58 (38.5%)	Cyclotron
^67^Cu	61.9	β − 0.4 (100%)	cyclotron
^89^Zr	78.0	Β ± 0.9 (100%)	cyclotron
^212^Pb	10.6	β − 0.6 (~80%); γ − 0.2 (44%); 0.08 (18%)	generator ^228^Th→^220^Rn→^216^Po→^212^Pb
^212^Bi	1.0	α − 6.0 (100%); β − 2.0 (100%); α/β ** = 0.67	generator ^228^Th→^224^Ra→^212^Bi
^213^Bi	0.7	α − 5.8 (97%); β − 1.4 (100%); α/β ** = 0.02	generator ^229^Th→^225^Ac→^213^Bi
^211^At	7.21	α − 5.9 (42)	cyclotron
^225^Ac	240.2	α − 5.7 (100%)	generator ^229^Th→^225^Ac
^223^Ra	273.6	α − 5.7 (100%)	cyclotron
^149^Tb	4.1	α − 4.0 (~80%)	cyclotron
^226^Th	0.5	α − 6.3 (~50%)	generator ^230^U→^226^Th
^227^Th	448.8	α − 6.0 (48%)	generator ^227^Ac→^227^Th
^89^Sr	1212	β − 1.5 (100%)	nuclear reactor
^153^Sm	46.3	β − 0.81 (100%)	cyclotron

* Percentage of quanta with the indicated energy value in the total amount of quanta of this type emitted by a given radionuclide; ** Ratio of the amount of quanta of different emission types.

These isotopes represent a standard being used for comparison with all other radionuclides [19]. The widespread use of ^131^I and ^90^Y in TRNT because of their suitable emission characteristics, facile and affordable production, and amenable chemical properties, which allow simple and stable attachment of radionuclides to carrier molecules. There are a number of published studies describing the efficiency of these radionuclides for clinical treatment of hematological and, more rarely, solid malignancies. Radionuclide ^131^I is relatively inexpensive and can be used in both visualization and therapy, with many years of successful implementation in the therapy of a number of malignant tumors, including thyroid cancer. A disadvantage of the application of ^131^I is stems from the ^131^I-tyrosine and free ^131^I in the blood flow of patients, and this event is especially sharply manifested upon fast biodegradation of molecules carrying ^131^I via endocytosis by tumor cells [20]. Additionally, γ-rays emitted by ^131^I can pose radiological hazard for patient family members and healthcare personnel. Injection of high doses of ^131^I-containing pharmaceuticals in some cases requires hospitalization and isolation of the patient. A radionuclide ^90^Y is a reasonable alternative to ^131^I for therapeutic applications. Its advantage stems from that its ionizing radiation hardly leaves the patients’ organism, and the healthcare personnel and family members bear much lower load of the radiation in comparison to that of ^131^I. Moreover, ^90^Y has a shorter half-life, nearly two-fold higher energy of β-particles and high retention in tumor cells. A higher price can be seen as a disadvantage [21]. Attempts for application of a number of radionuclides in targeted therapy are described in the literature [22,23].

It should be noted that some radionuclides do not require the use of carrier molecules due to their chemical properties. Such radionuclides exhibit tropism for certain tissues and thus effectively accumulate in them, allowing for targeted therapeutic effect. Such radionuclides include isotopes ^223^Ra, ^89^Sr, ^153^Sm accumulating in the bone tissue, and ^131^I in the thyroid gland.

The use of ^131^I in clinical practice without any carrier molecules lasts for more than 70 years. In the beginning of the XX century a method for hyperthyroidism treatment with ^131^I was proposed and implemented, and starting from 1942 this innovation has been successfully applied in different clinics around the world [24]. Since 1946 ^131^I has been successfully applied as an effective means for diagnosis and treatment of bone metastases of differentiated thyroid carcinomas of papillary and follicular types [25]. Today it is known that iodine accumulation by thyroid cells though the action of Na-I-symporter (NIS), a protein actively capturing iodine in thyroid cells [26]. Following the discovery of NIS, an idea of pharmacological correction of NIS expression appeared; the higher the level of NIS in the membrane, the more the efficiency of ^131^I accumulation in cancer cells. It is currently known that NIS increase in the tissues can be achieved though the use of some agents, such as early growth response protein 1 from nonthyroid malignancies (Egr1, early growth response 1) [27], TSH-dependent transcription factor of NIS (NTF-1, NIS TSH-responsive factor-1) [28], redox factor-1 (Ref-1) [29] box-8 (Pax-8) [30] *etc.* Despite the achieved progress in this direction, these proteins are at present not used in clinical practice. In the last five years a novel approach was developed for NIS-dependent radioiodine therapy of non-thyroid malignancies. This method is based on a significant increase in the speed of radioactive iodine uptake by tumor cells after transfection of these cells with the NIS gene using viral and non-viral vectors [24,31].

Drugs that include ^89^Sr, ^153^Sm, and ^223^Ra, also without any carrier molecules, can be used in palliative therapy of ostealgia in patients with multiple painful bone metastases in various forms of cancer, including prostate cancer, breast cancer, *etc.* Based on ^223^Ra a pharmaceutical of radium dichloride was developed and clinically tested [32]; it is currently utilized for therapy of bone metastases in disseminated prostate cancer [33]. Notably, unlike ^153^Sm and ^89^Sr, the ^223^Ra-containing drug is an alpha-emitter, and ^219^Rn, ^215^Po, ^211^Bi in the series of its derivatives are also alpha-emitters with short half-lives, with total decay energy ^223^Ra equaling 28.2 MeV. The ^223^Ra-based drug possesses a high rate and selectivity of accumulation in target tissues, so that only 20% of the initial amount of ^223^Ra persists in the blood within 15 minutes after intravenous injection, with about 60% of the isotope localized in the bones and 40% excreted without significant accumulation in non-target organs (heart, liver, spleen) [34].

As described previously, in TRNT in some cases alpha-emitting radionuclides are used, which are characteristic for high relative biological effectiveness, linear energy transfer and the energy of radioactive decay. For a number of isotopes long chains of daughter products of decay are known (^225^Ac, ^223^Ra, ^227^Th, ^226^Th), and their daughter isotopes are also alpha-emitters that can reduce the required amount of drug compared to beta-emitters. These advantages make alpha-emitting radionuclides particularly attractive for the therapy of non-solid tumors and micrometastases [35]. However, despite the obvious advantages, there are objective reasons hindering the use of alpha-emitting radionuclides in TRNT. The most critical of those are (1) the presence of beta-emitters among daughter products, which cause radiation injury to adjacent tissues (for example, ^211^Pb and ^207^Tl, which are products of decay of ^223^Ra; ^213^Bi and ^209^Pb–^225^Ac); (2) the presence of long-lived isotopes among daughter products, resulting in the necessity for patient isolation (for example, beta-emitting ^207^Bi, with a half-life of 33 years, daughter product ^211^At); (3) potential volatility of the chemical bond with the carrier molecule (in particular, similarity in the chemical properties of ^211^At and iodine are often considered to be advantages of this nuclide; however, *in vivo* the stability of the majority of compounds labeled with astatine, is poor), resulting in a decrease in delivery orientation of the isotope to the target organs and tissues; and (4) potential tendency to self-radiolysis, partial drug destruction due to its radioactivity.

## 4. Choice of Carrier Molecule

It is clear that carrier molecules for targeted therapy ought to meet the following criteria.

(1) The carrier molecule must possess high affinity and specificity for the target.
(2) The carrier molecule should not be toxic or immunogenic, with a preferable LD_50_ value of greater than 1.5 g per kg of body weight.
(3) The carrier molecule has no resistance to self-radiolysis and well-preserved both under storage conditions and upon contact with biological liquids.
(4) Production of the carrier molecules with sufficient chemical purity ought to be simple and cost effective.
(5) The carrier molecule has binding affinity to a variety of radionuclides. Specific chemical modifications are preferred to involve a minimum number of reactions.

The main carriers of radionuclides in therapy are antibodies and peptides, though attempts have been made to exploit liposomes and bisphosphonate molecules, dextran, *etc.* as carrier agents [36]. Owing to the high specificity, monoclonal antibodies and their fragments are among the most suitable carrier molecules for TRNT [37]. Most malignancies are characterized by the overexpression of discriminative antigens predominant in tumors. Monoclonal antibodies with the affinity to the cancer-specific antigen is produced by means of well-established hybridoma technology. Antibodies can be bound to radionuclides by a direct bioconjugation or by using a bifunctional chelate. Although this technology has been proved very useful and efficient, its drawbacks include large size, as exemplified by the smallest immunoglobulin G (IgG) weighing 150 kDa, and slow kinetics in comparison to peptides [38]. Excessive expression of numerous peptide receptors on the surface of human tumor cells against that in normal tissue cells makes analogues of signal peptides effective for the role of carrier molecules for TRNT. The peptide carriers are most often used in the therapy of neuroendocrine tumors (predominantly somatostatin analogues [39]), as well as in the treatment of rectal cancer (peptides to antigens EphA2, RNF43-721, ABT-737) [40].

The other transport carriers have been also employed for target delivery of radionuclides. Among these, liposomes can transport radionuclides to highly vascularized tumors, harbored predominantly in the liver or spleen. The radionuclides can be encapsulated either in the cavity space of a liposome or between the layers [41]. Such polymers as dextran, chitosan, polylactide or polylactide glycolide, *etc.*, are also employed as the transport carriers in TRNT [41]. Dextran, being one of the representatives of the transport carriers, is a branched polysaccharide, with glucose molecules as monomers. Dextran is used for passive accumulation of radionuclides in vascularized tumors predominantly in the liver and spleen, where the accumulation mechanism is similar to that of liposomes [36]. The accumulation of these transport carriers in the tumor is primarily due to an increased permeability of the tumor blood vessels featuring abnormally variable and large mean-size fenestrations (100–200 nm). Biomolecular polymers and nanoparticles sized in excess of 10 nm extravasate into the tumor interstitial space and are retained in the tumor interstitium due to the dysfunctional lymphatic drainage. This effect was called the enhanced permeability and retention (EPR) effect in the literature [42]. Another carrier type, bisphosphonates, are natural biologically-stable analogues of pyrophosphates. They inhibit bone resorption by preventing activation of osteoclasts and contribute to the bone formation though the stimulation of osteoblast differentiation. Bisphosphonates are successfully used for radionuclide delivery because they effectively bind to hydroxyapatite in the regions of active osteogenesis, which favors their accumulation in the vicinity of bone tumors [43].

## 5. Selection of a Target Antigen of Tumor Cells

The choice of an optimal antigen exposed on the membrane of tumor cells is crucial for therapy and diagnostics. An ideal TRNT antigen must meet the following criteria:

(1) Even expression and distribution over the entire cells surface of malignant tumors;
(2) Low or negligible expression in normal cells to minimize the side effects;
(3) Detainment in the cancer site, with no leakage to the blood flow.

More detailed requirements for the selection of target antigens of tumor cells for clinical purposes are described in a recent review [38].

Target antigens are usually represented by macromolecules localized on the surface of cell membrane of tumor cells. They are easily accessible from the blood and extracellular fluid and often possess hematopoietic clusters of differentiation (CD), which are normally expressed during the maturation of various types of blood cells. Suitable target antigens commonly include cell surface glycoproteins (mucins, GPA33, NG2), enzymes (PSMA, CAIX), glycolipid (GD2), stromal components (FAPα), blood vessel elements (integrins, VEGFR, fibronectin B), and signal transduction molecules (GFR, EGFR, HER2) [44]. None of the existing target antigen is ideal for TRNT, although several types of tumors present nearly ideal targets. For example, CD20 and CD22 represent target antigens for lymphoma treatment [45], CD33 and CD45—for acute myeloid leukemia [46], PSMA [47] and fibronectin B [48]—for solid tumors, and GPA33—for colorectal cancer [49].

## 6. Targeted Radionuclide Therapy of Hematological Malignancies

The application of targeted radionuclide therapy for the treatment of hematological malignancies is in many aspects an effective approach due to the following reasons: (1) First of all, many cancer cell lines express specific surface antigens, which are not present in the other tissues of the organism; (2) At present time, a rich choice of high-quality antibodies against these antigens are available; (3) Leukemias and lymphomas are extremely sensitive to ionizing radiation [50]. Moreover, the availability of transplantation technologies for bone marrow cells allows increasing the irradiation dose. This is particularly important on application of the autotransplantation stem cell rescue (SCR) approach, when the irradiation at high doses follows native stem cells picking from the patient, with their subsequent use after a therapy course to replenish the largely depleted pool of stem cells. In clinical practice, radionuclide therapy of hematological malignancies often utilizes anti-CD20 antibodies, and these are applied for the production of conjugates with radionuclides ^131^I or ^90^Y. The overall therapy efficiency of deployment these pharmaceuticals is high, reaching 60%–80%, with a complete remission rate of 15%–40%. The median of complete remission duration for this TRNT is about 1–2 years, and 15%–20% of cases demonstrate a sustained remission lasting up to 10 years and longer [19]. Similarly, TRNT of the hematopoietic system makes use of antibodies against antigens CD33, CD45, and CD66 [51]. Promising targeting agents for preclinical and clinical studies include antigens CD5 in the therapy of chronic lymphocytic leukemia [52], CD30 and ferritin—for Hodgkin’s lymphoma treatment [53] and CD25—for the therapy of acute T-cell leukemias and lymphomas [54].

## 7. Targeted Radionuclide Therapy of Solid Tumors

The clinical efficiency of the existing TRNT of solid tumors remains low. The extreme resilience of solid tumor cells to the effects of ionizing radiation is at the heart of this problem and represents the major challenge of TRNT. This resilience is due to the uneven distribution of the radiopharmaceuticals in the tumor body, where the major portion is accumulated in the periphery. Cells located in the tumor parenchyma receive small ionizing radiation doze from the alpha- or beta-emitters localized at the periphery. In addition, a single cell is known to be more susceptible to the ionizing radiation that a monolayer of the same cells, and this is more susceptible that the cells in 3D matrix of scaffolds. It is also known that cancer cells located in tumor parenchyma are exposed to hypoxic conditions, which hampers generation of reactive oxygen species, which are characterized by the highest damaging potential.

**Figure 3:**
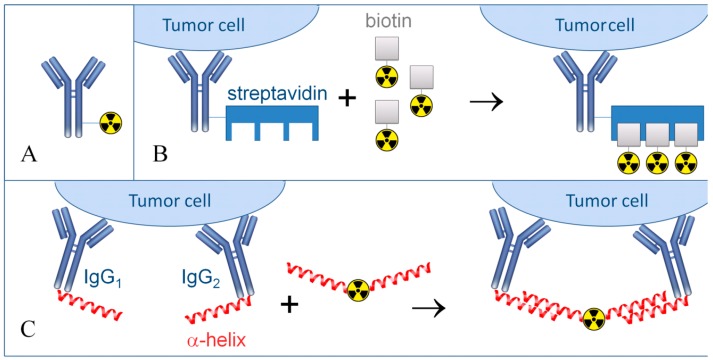
Schematic representation of conventional and pre-targeted radionuclide therapy. (**A**) Conventional targeted radionuclide therapy is realized only using monoclonal antibodies or other carrier molecules directly conjugated with a radionuclide; (**B**) Multi-step pre-targeted radionuclide therapy, “amplifier” mode. At first, antibodies conjugated with streptavidin are used and following antibody binding radiolabelled DOTA-biotin is introduced. It is presumed that each streptavidin molecule binds to four molecules of radionuclide-labeled biotin; (**C**) Multi-step pre-targeted radionuclide therapy, mode for specificity increase.

Multi-step pre-targeted radionuclide therapy is presently utilized to solve this issue, which is capable of providing a significant increase of the therapeutic selectivity and enhance the tumor radiation exposure (Figure 3).

It is therefore worthwhile to present several cases of successful TRNT of solid tumors. For example, the clinical practice of targeted radionuclide therapy of metastases of hepatic colorectal carcinoma involved the use of anti-CEA antibodies, which were employed to produce a conjugate with ^131^I isotopes. The median survival time in such TRNT was approximately 68 months, with the median of complete remission duration of 18 months [55]. The treatment of castration-resistant prostate cancer, where antibodies against PSMA antigen were utilized to produce a conjugate with ^177^Lu radionuclides represent another successful approach, achieving the median survival time up to 10 months [56]. In stage IV melanoma treatment antibodies against NG2 were used with a radionuclide ^213^Bi. In 50% of patients, a long-lasting stable course of the disease was registered, while 14% had a partial remission [57]. Metastasizing melanoma therapy included anti-NG2 antibodies with radionuclide ^213^Bi, and the median survival time in that targeted radionuclide therapy was increased by nine months [58].

The first step assumes the use of antibodies against one or several antigens, conjugated with an α-helix, for example, from phosphorodiamidate morpholino oligomer. After antibody binding, radionuclides are introduced, which are conjugated with α-helices complementary to antibody-bound α-helices.The TRNT of solid neuroendocrine malignancies effectively takes advantage of applying selective peptides. For example, in clinical practice the targeted radionuclide therapy of pancreatic tumor is realized using peptide DOTATATE, a somatostatin analog, conjugated with a radionuclide ^177^Lu. Over a period of 20 months, 12% of patients exhibited a complete remission, with a partial remission in 27% of patients and a stable disease course without complications in 46% cases [59]. When peptide DOTATOC and radionuclide ^90^Y were utilized in pancreatic tumor treatment, 4% of patients demonstrated a complete remission, 23% had a partial remission, and a stable disease course without complications was registered in 62% of patients [60]. The application of an equivalent pharmaceutical in the treatment of gastroneuroendopancreatic tumors resulted in a complete remission in 5% of patients, a partial remission in 18% of patients and a stable disease course without complications in 69% cases [61]. The overall progress achieved in the application of peptide-mediated tumor radiotherapy is reviewed in the literature [62,63,64].

## 8. Determination of the Dose Load in TRNT

In documents regulating the radiation impact on human body, the concept of critical organ is applied as an organ or tissue, a body part, the irradiation of which can cause the highest damage to the health of a biological object or its progeny under certain conditions. In case of internal irradiation the concept of a critical organ seems to be more complicated than in external exposure. The values of threshold damaging doses for different organs/tissues and the distribution of radionuclides in organs/tissues (tropism of radionuclides) are of major importance. The most radiosensitive organs are lymphoid organs, red bone marrow, gonads, and the small intestine, which are damaged upon radiation at the lowest doses [65]. It should be noted that different subjects possess significant individual levels of radiosensitivity in various organs, which can be nearly up to 20% of the values regarding the dose load [66]. The ability of preferential accumulation in certain organs allows defining osteotropic (radionuclides selectively deposited in the bones—radium, strontium, barium, calcium), hepatotropic (selectively accumulated in organs rich in reticuloendothelial elements—lanthanum, cerium, promethium, praseodymium, as well as actinium, thorium, some compounds of plutonium) and radionuclides evenly distributed throughout the body (isotopes of cesium, potassium, sodium, rubidium, hydrogen, carbon, nitrogen, and other elements, including polonium). Separate groups include radioactive isotopes of iodine, which are selectively accumulated in the thyroid gland, and poorly reabsorbed radionuclides, which cause local processes that are localized depending on their modes of intake.

TRNT is implemented with the assessment of radiation doses to healthy tissue and organs. One of the methods for determination of the predicted absorbed dose for organs and tissues is the modeling method used, for example, for assessing the exposure upon application of radium-223 chloride [67]. The most objective assessment is based on extensive statistical material and operates such indicators as the manifestation of long-term side effects and the overall survival of patients; however, a number of individual factors specific for each individual patient are necessary to be taken into account in determining the dose of a drug for TRNT [68]. The task of providing an individual approach to the dosing of a drug for TRNT is aided by the conduction of individualized dosimetry studies. Pre-treatment dosimetry is implemented to estimate the radiation load for tissues and organs of a patient, which uses the carrier molecule; however, the radionuclide used in the therapy is replaced with a short-lived gamma- or β-emitting isotope. In some cases an isotope of the same element that is used in therapy is used as a label—for example, ^124^I, a source of positron emission is used as a label for the therapeutic isotope ^131^I [69,70]. Another widely used therapeutic radionuclide ^90^Y is used along with ^87^Y [71], though this application is limited by the osteotropism of the daughter product of radioactive decay ^87^Sr. The medications of ^90^Y are often accompanied by the use of gamma-emitting ^111^In having a stable decay product ^111^Cd and a half-life of 2.8 days [14] The application of pre-treatment dosimetry allows the creation of a radiation dose record and determine the required dose of the drug individually [72].

## 9. Available Commercial Pharmaceuticals for Targeted Radionuclide Therapy

Currently, several pharmaceuticals for TRNT of tumors are being introduced into clinical practice (Table 2) [73]. The radionuclide component of the most known drugs is featured by ^90^Y or ^131^I. These pharmaceuticals are mostly effective against hematological malignancies. Most preparations are extremely costly, sometimes having noncompetitive prices, and some of them, like Bexxar (produced in 2003–2014), were taken out of production because of commercial reasons.

Zevalin^®^ (Ibritumomab tiuxetan) is the first pharmaceutical for radio-immunotherapy commercially available worldwide. In February 2002, the U.S. Food and Drug Administration (FDA) approved it for the therapy of recurrent and resistant forms of low-grade follicular B-cell non-Hodgkin’s lymphoma. CD20 and ^90^Y were used as an antigen and a radionuclide, respectively [74]. The results of phase III of clinical trials for Zevalin (90Y-ibritumomab tiuxetan) undertaken in 2001–2005 are presented in the study [75], which is an update of a previous work of the same working group published in 2008 [76]. An international randomized study involved 414 patients with CD20-positive stage III or IV follicular Non-Hodgkin's lymphoma. Mandatory inclusion criteria were complete or unconfirmed complete remission after first-line therapy (complete response (CR), unconfirmed CR (CRu), or partial response (PR) after first-line induction treatment). Patients from the therapeutic group received ^90^Y once at a dose of 14.8 MBq/kg (but not exceeding 1.184 GBq). On an eight-year interval progression-free survival (PFS) of 41% was demonstrated compared with 22% for patients in the control group. Long-term side effects were not reported.

**Table 2:** Drugs for radio-immunotherapy.

Commercial Name (Other Names)	Antigen/Radionuclide	Disease	Clinical Trial Status
Zevalin (^90^Y–ibritumomab tiuxetan)	CD20/^90^Y	non-Hodgkin‘s lymphoma	Approved by FDA
Bexxar ( ^131^I–tositumomab)	CD20/^131^I	non-Hodgkin‘s lymphoma	Approved by FDA
Oncolym (^131^I–Lym 1)	HLA-DR10/^131^I	non-Hodgkin‘s lymphoma, chronic lymphocytic leukaemia	Phase III
Lymphocide (Epratuzumab)	CD22/^90^Y	non-Hodgkin‘s lymphoma, chronic lymphocytic leukaemia, immune diseases	Phase III
Cotara (^131^I–chTNT–1/B)	DNA/^131^I	glioblastoma, anaplastic astrocytoma	Phase III
Labetuzumab (CEA–Cide)	CEA/^90^Y or ^131^I	breast, lung, pancreatic, stomach, colorectal carcinoma	Phase III
Theragin (Pemtumomab)	PEM/^90^Y	ovarian, gastric carcinoma	Phase III
Licartin (^131^I–metuximab)	(Hab18G/CD147)/^131^I	hepatocellular carcinoma	Phase II
Radretumab (^131^I–L19)	Fibronectin/^131^I	hepatological malignancy, refractory Hodgkin‘s lymphoma, non-small cell lung cancer, melanoma, head and neck carcinoma	Phase II
PAM4 (^90^Y–clivatuzumab tetraxetan)	MUC1/^90^Y	Pancreatic adenocarcinoma	Phase III
Xofigo (^223^Ra dichloride)	–/^223^Ra	metastatic castration-resistant prostate cancer	Approved by FDA
Lutathera (^177^Lu–DOTA–Tyr^3^–Octreotate)	SST/^177^Lu	metastatic GastroEnteroPancreatic NeuroEndocrine Tumors	Phase III
^131^I–MIBG	norepinephrine (NE)/^131^I	neuroblastoma, Pheochromocytoma, Paraganglioma	Phase III

Lymphocide^®^ (Epratuzumab) is a pharmaceutical, which is being tested in phase III of clinical trials. CD22 and ^90^Y were used as an antigen and a radionuclide, respectively. The pharmaceutical was designed for the therapy of B-cell non-Hodgkin’s lymphoma and chronic lymphocytic leukemia [77]. Theragyn^®^ (Pemtumomab)—a pharmaceutical also known as Theragyn or HMFG1; it is being tested in phase III of clinical trials. Monoclonal mouse antibody against mucin MUC1 was combined with radionuclide ^90^Y. Glycoproteid mucin is expressed on the surface of epithelial cancer cells, including ovarian, gastric, breast, and lung cancer types [78]. PAM4 (^9^^0^Y-clivatuzumab tetraxetan)—pharmaceutical characterized by a high efficiency against pancreatic cancer is currently in phase III of clinical trials. It does not accumulate in normal tissues and exhibits limited accumulation in the other types of tumors. ^90^Y, and antigen CA19–9 associated with pancreatic adenocarcinoma were deployed in this case [79]. The results of phase I clinical trials of efficiency of a drug based on 90Y-clivatuzumab tetraxetan against pancreatic carcinoma were published in [14,72]. The studies involved 42 and 21 patients, respectively, aged 33–86 years, at stages III-IV of the disease. During the study the patients received the ^90^Y-based drug three times at a dose of 6.5–15 mCi/m^2^ [14] and 15–25 mCi/m^2^ [72]. The survival median was 7.7 in the studies (six months for patients at stage IV and 19.6 months for patients at stage III) and 4.3 months, respectively.

CEA-Cide (Labetuzumab) is under preparation for phase III of clinical trials. The antibodies used possess affinity for carcinoembryonic antigen (CEA). CEA is expressed in 90% of colorectal cancer cases [55]. Labetuzumab can incorporate either ^90^Y or ^131^I as the radionuclide component. Bexxar^®^ (Tositumomab) is a ^131^I radionuclide containing drug, also known as ^131^I-Tositumomabor, was approved by FDA in 2003.

Bexxar is trophic to the CD20 receptors on the surface of tumor cells. It was reported to have efficacy for the therapy of B-cell non-Hodgkin’s lymphoma, diffuse B-cell lymphoma, and multiple myeloma [80]. Randomized phase III clinical trials (ClinicalTrials.gov Identifier NCT00006721), published in the Journal of Clinical Oncology [81] involved 532 patients with biopsy-proven, untreated, bidimensionally-measurable bulky stage II or stage III–IV FL (grade 1, 2, or 3) expressing CD20. Every patient received six cycles of treatment with cyclophosphamide, doxorubicin, vincristine and prednisone (CHOP) chemotherapy at three-week intervals. After that patients from the control group received 6 doses of rituximab (CHOP-R), while the patients from the experimental group followed by consolidation with tositumomab/iodine I-131 tositumomab radioimmunotherapy (RIT). The results of the study on a two-year interval showed a progression-free survival (PFS) of 76% and 80% for CHOP-R and CHOP-RIT arms, correspondingly. The two-year estimate of the overall survival (OS) was 97% on the CHOP-R arm and 93% on the CHOP-RIT arm. In another randomized study (ClinicalTrials.gov Identifier NCT00329030), published the same year [82], the efficiency of radioimmunotherapeutic approach (dose of 5 mCi of ^131^I-tositumomab resulting in the absorbed dose of 0.75 Gy in seven days) was compared with the effectiveness of rituximab (375 mg/m^2^) for relapsed diffuse large B-cell lymphoma, conducted in both cases according to the same protocol. Both drugs were administered 14 days before the start of chemotherapy and rituximab was administered again seven days before chemotherapy. The study involved 224 patients, 111 of which received a course of radioimmune therapy. Two-year progression-free survival (PFS) rates, the primary end point, were 48.6% and 47.9% for rituximab and ^131^I-tositumomab, respectively. The two-year overall survival (OS) rates were 65.6% and 61%. Thus, the results obtained for rituximab and ^131^I-tositumomab are statistically indistinguishable [83], though an improvement of PFS was registered in patients with normal serum β2M, whereas in patients with high serum β2M, the PFS was similar by arm.

Oncolym^®^ (^131^I-Lym-1) is a pharmaceutical being evaluated in phase III of clinical trials. It binds to human leukocyte antigen (HLA) [84] DR10, which is exposed only on the surface of B-lymphocytes, such as cancer B-lymphocytes. Cotara^®^ (^131^I-chTNT-1/B)—pharmaceutical based on a chimeric monoclonal antibody is in phase III of clinical studies. Its molecules bind to the complex DNA-histone H1, wherein ^131^I is used as the radionuclide component. This pharmaceutical is efficient against anaplastic astrocytoma, brain glioblastoma, hepatocellular carcinoma, colorectal cancer and glioma [85]. Licartin^®^ (^131^I-metuximab) is a pharmaceutical composed of a monoclonal mouse antibody Hab18 F(ab’) trophic to an antigen Hab18G/CD147 of hepatocellular carcinoma and radionuclide ^131^I. The monoclonal antibody itself possesses cytotoxic activity and is potentially capable to inhibit metastasis of hepatocellular carcinoma through blockade of matrix metalloproteinase synthesis. In a complex with ^131^I, this pharmaceutical exhibits enhanced potency due to tis direct effect on tumor cells. The drug is in phase II of clinical trials, although it has already gained approval of the China Food and Drug Administration (CFDA) for patients with inoperable or recurrent hepatocellular carcinoma [86]. Radretumab^®^ (^131^I-L19) is a pharmaceutical being tested in phase II of clinical trials and represents monoclonal antibodies specific for isoforms originating upon alternative splicing of fibronectin and tenastin-C, combined with a radionuclide ^131^I. The drug is effective against non-small cell types of lung cancer and hematological malignances [87]. Xofigo is a trade mark of radium-223 dichloride produced by Bayer HealthCare Pharmaceuticals Inc. The drug was approved by the FDA in May 2013 for use in the therapy of metastatic castration-resistant prostate cancer [88]. Due to osteotropism of radium Xofigo accumulates in the bone tissue [34]. Peculiarities of *Ra* localization in the bone matrix condition its low mobility, thus, despite the long decay chain the drug has low toxicity for the surrounding tissues. A review of main clinical and preclinical studies for drugs containing ^223^Ra is presented in the works [34,89]. The most large-scale clinical trials using radiul-223 cloride Xofigo™ took place from 2008 to 2011 (ClinicalTrials.gov Identifier NCT00699751, A Phase III Study of Radium-223 Dichloride in Patients With Symptomatic Hormone Refractory Prostate Cancer With Skeletal Metastases (ALSYMPCA)) and were published in [90]. International, placebo-controlled, randomized studies included 921 patients with bone-metastatic, castrate-resistant prostate cancer. The patients were subjected to six-time injection of Xofigo™ at a dose of 50 kBq/kg with an interval between injections of four weeks. The overall survival was 14.9 months, compared to 11.3 months for placebo.

The drug Lutathera (^177^Lu-DOTA-Tyr3-Octreotate) is available for clinical trials by Advanced Accelerator Applications. The specificity of Octreotate binding with SSTRs determines its effectiveness in the therapy of NET. Currently the drug is being successfully tested in phase III clinical trials (ClinicalTrials.gov Identifier NCT01578239), and the successful results of randomized studies in 230 patients were published in September 2015 [91]. The study involved 230 patients with Grade 1–2 metastatic midgut NETs. In the studies the patients were injected four times with Lutathera with 8-week intervals at an activity of 7.4 GBq. By the time of results presentation, the median progression-free survival (PFS) for Lutathera has not been achieved, while in the control group (drug Octreotide LAR 60 mg every 4 weeks) the median PFS was 8.4 months (mortality in the group of Lutathera and the control group by the date of announcement of the results was 23 and 67 cases, respectively. Starting from April 2015, the FDA allowed the application of Lutathera in the treatment of inoperable progressive midgut NETs. The pharmaceutical of ^131^I-MIBG (metaiodobenzylguanidine) was originally tested for NET treatment, however, the highest interest was connected with the ability of MIBG to penetrate through cell membranes via norepinephrine(NE)-transporter, which is actively expressed in neuroblastoma cells [92]. One of the major obstacles for the wide application of the ^131^I-MIBG-based drug in clinical practice is its toxicity for the tissues of the thyroid gland and liver, which, however, can be reduced by means of prophylactic intake of medications with potassium iodide and potassium perchlorate [93]. In 2011–2015 the drug ^131^I-MIBG was tested for identification of the possible toxicity at high (15–18 mCi/kg) doses in combination with vincristine and inhibitor of topoisomerase irinotecan for resistant/relapsed neuroblastoma (ClinicalTrials.gov Identifier NCT01313936). The testing was performed with the participation of the University of California, supervised by Steven G. DuBois. A total of 32 people aged 2 to 30 years took park in the study. All patients received a five-day course of chemotherapy with irinotecan and a one-dose injection of ^131^I-MIBG on the second day of chemotherapy. The therapeutic effect was achieved in nine cases, though two cases exhibited a marked side effect of dehydration. Similar studies, in which the use of beta-emitting ^131^I-MIBG was combined with chemotherapy using histone deacetylase inhibitor vorinostat were described in the publication [94]. The studies were conducted in 27 patients aged up to 30 years, who were subjected to a two-week chemotherapy course including injection of ^131^I-MIBG at a dose of 8–18 mCi/kg on the third day of therapy. The therapeutic effect was observed in 28% cases, and the combination of 180 mg/m^2^/dose vorinostat and 18 mCi/kg ^131^I-MIBG was recommended for transition to stage II clinical trials (therapeutic effect of 67% in 6 patients). The overall retrospective analysis of the efficiency of ^131^I-MIBG therapy in the treatment of recurrent and chemotherapy-resistant neuroblastoma is provided in the study [95], testifying a partial or complete efficiency of the drug in 27% cases given the 24-month overall survival of 47% (65% and 39% in the case of recurrent and chemotherapy-resistant neuroblastoma, respectively). Currently the drug is in phase III of clinical trials conducted by the New Approaches to Neuroblastoma Therapy Consortium (ClinicalTrials.gov Identifier NCT02035137) and Tehran University of Medical Sciences (ClinicalTrials.gov Identifier NCT00798148).

## 10. Conclusions

Currently, significant progress has been made in targeted radionuclide therapy due to the development of molecular and cell biology, immunology, radiation and medical biophysics, nuclear physics, chemical technology, and other related disciplines. To date, a large number of tumor cell antigens suitable for application in targeted radionuclide therapy have been introduced and characterized. A wide variety of carrier molecules, mainly antibodies and some peptides, have been constructed and synthesized. A variety of α-, β-, and Auger electron-emitting radionuclides have been introduced, along with the technologies for their synthesis, isolation, and purification. These radionuclides can now be reliably attached to carrier molecules and target-delivered to practically all types of tumors in the human organism upon intravenous and intracisternal injections, owing to the developed techniques and protocols. Additionally, deposition and dosage of these radionuclides can be measured and controlled. These advances lead to a novel theranostic approach towards personalized treatment regimens and provide an overall increase of the therapeutic efficiency. Overall, the key fundamental concepts and technology platform of the targeted radionuclide therapy have been established. At the same time, tangible therapeutic outcomes have been demonstrated only in the therapy of hematological malignancies. The efficiency of TRNT of solid tumors, especially large-sized tumors is notoriously low.

In order to achieve a high therapeutic index of TRNT of solid tumors, the key problem of accumulation of high doses of ionizing radiation in tumor tissue cells must be solved. Reliable destruction of radiosensitive tumors requires the absorbed doses to be in the range of approximately 3000–5000 cGy, while elimination of solid tumors with the highest radioresistance, such as thyroid tumors therapy needs up to 10,000 cGy. At the same time, the absorbed doses in organ tissues, including kidneys, lungs, colon mucosa bone marrow should not exceed 2000 cGy, 1500 cGy, 250 cGy and 100 cGy, respectively [19]. It should be noted that in TRNT the effect of ionizing radiation on normal tissues takes place not only during the “trip” of a pharmaceutical through the organism from the injection point to the tumor, but also in the result of metabolism of the complex pharmaceutical-antigen. The metabolism of this complex can either enhance the antitumor effect through radionuclide deposition, or inhibit it due to radionuclide elimination from the cell. Some antigens, including CD5 and PSMA are rapidly internalized in cancer cells, leading to catabolism of the carrier-target complex and, specifically, radionuclide separation from the carrier molecule followed by its efflux from the cell. On the other hand, the uptake rate of such antigens as GPA33 and CD20 is slow, leading to an increased effective period of radionuclide immobilization on cancer cells. The disintegration of the carrier molecule and concomitant radionuclide release from the cell leads not only to the reduction of the therapeutic efficiency, but also causes normal tissue overexposure to the ionizing radiation, especially the liver and kidneys. It is, therefore, reasonable to implement dosimetry and practice symptomatic application of radioprotective adaptogenic drugs ineffective in tumor tissues, which combine radioprotective and hepatoprotective properites [96]. Guanosine [97], inosine [98], IMP [98], and GMP [99] represent such drugs for the liver. By all means, the development and identification of novel more efficient multifunctional radioprotective drugs can offer an alternative solution to the problem of high absorbed doses in normal tissues.

To summarize, TRNT is an important area of medicine, with a great potential to be unveiled, where multidisciplinary approach is deemed essential. Thus, the age of inventing the “sling, bow and firearms” in targeted radionuclide therapy is finished, and it is time to work on “high-precision rocket weapons and lasers”.
